# Assessment of the Efficacy of Telephone Medicine Consultations in Trauma and Orthopaedics During COVID-19 Using the Ashford Clinic Letter Score

**DOI:** 10.7759/cureus.13871

**Published:** 2021-03-13

**Authors:** Marjan Raad, Sebastian Ndlovu, Daniel Neen

**Affiliations:** 1 Trauma & Orthopaedics, Darent Valley Hospital, Dartford, GBR

**Keywords:** covid-19, telemedicine (tm), ashford clinic letter score

## Abstract

During the coronavirus disease 2019 (COVID-19) pandemic, our aim was to protect staff and patients, therefore, face-to-face clinics were converted to telephone clinics. We retrospectively compared two groups of patients: those seen in traditional clinics pre-COVID-19 and those who had telephone clinics during the pandemic. The mean Ashford Clinic Letter Score (ACLS) for the face-to-face clinic letters was 6.7, and the letters from both groups of telemedicine appointments scored better; the first group scoring 7.1 and the second 7.45. The pandemic allowed us to show that telephone clinics are effective and can be superior to traditional clinics in a specific set of patients.

## Introduction

In the past few years, telemedicine/telehealth has become more acceptable and is present in more than 125 countries, even though, in the past, telemedicine has had challenges and some resistance despite great potential [1]. As the coronavirus disease 2019 (COVID-19) pandemic spreads swiftly, international health organisations, governments and hospitals are grappling to contain the spread [2]. Adaptation is a necessity during this crisis. Telemedicine has taken centre stage across a variety of medical specialities and appears to provide solutions to some of the problems faced. Remote consultations with telephone clinics, virtual fracture clinics and video consultations have been adopted in many trauma and orthopaedic teams [3-4].

As telemedicine was becoming an integral part of our everyday duties at work, we wanted to determine whether telephone clinics are safe, effective, and more efficient compared to face-face clinics regarding making a diagnosis, organising appropriate investigations and making an appropriate treatment plan for the patient using the Ashford Clinic Letter Scoring System (ACLS). This tool has proven to be reliable, reproducible, and concise, which aids in objectively assessing and auditing the quality and efficacy of consultations [5].

## Materials and methods

In this study, we retrospectively compared two groups of patients: those seen in traditional clinics pre-COVID-19 and those who had telephone clinics during the COVID-19 pandemic. We collected 60 fracture clinic letters from a one-week period prior to the COVID-19 pandemic in March 2020. The clinical correspondence included consultations carried out by the consultant, middle-grade trainees and associate specialists. During this period, the whole service was face-to-face clinics, and this helped reduce selection bias. Cases included a mixture of new trauma referrals and trauma follow-ups, which included operative and non-operative patients.

During COVID-19, all clinics were converted to telephone clinics unless a patient required a plaster change or wound check. We then collected 60 fracture clinic letters from telephone consultations from a one-week period at the start of the pandemic in April 2020. Lastly, we collected another 60 fracture clinic letters from telephone consultations from a one-week period one month later in May 2020. We carried out the third set of collection of clinic letters to assess two things: was there a decrease in the number of new patients being referred to the fracture clinic during the pandemic and were clinicians becoming more confident to discharge and make final plans for patients over the phone.

**Figure 1:**
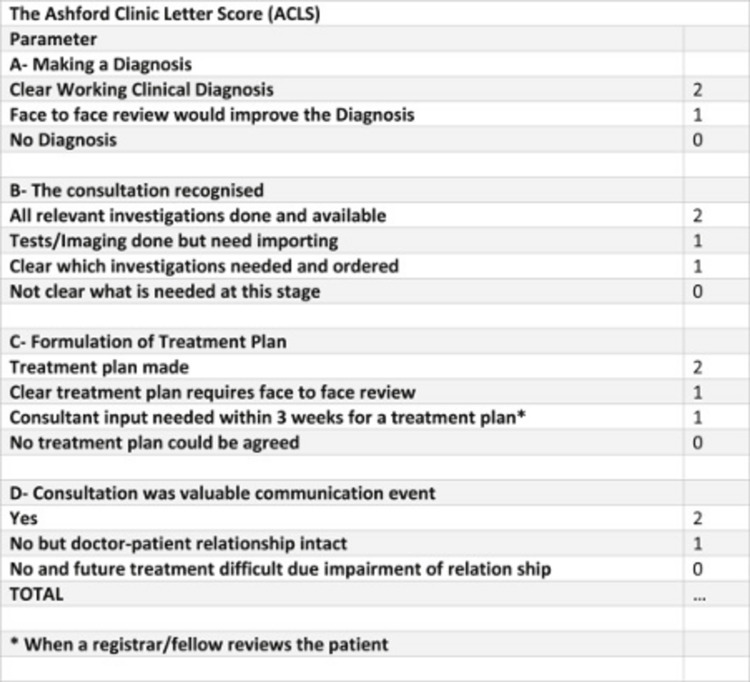
The Ashford Clinic Letter Score (ACLS): components and scoring

**Figure 2:**
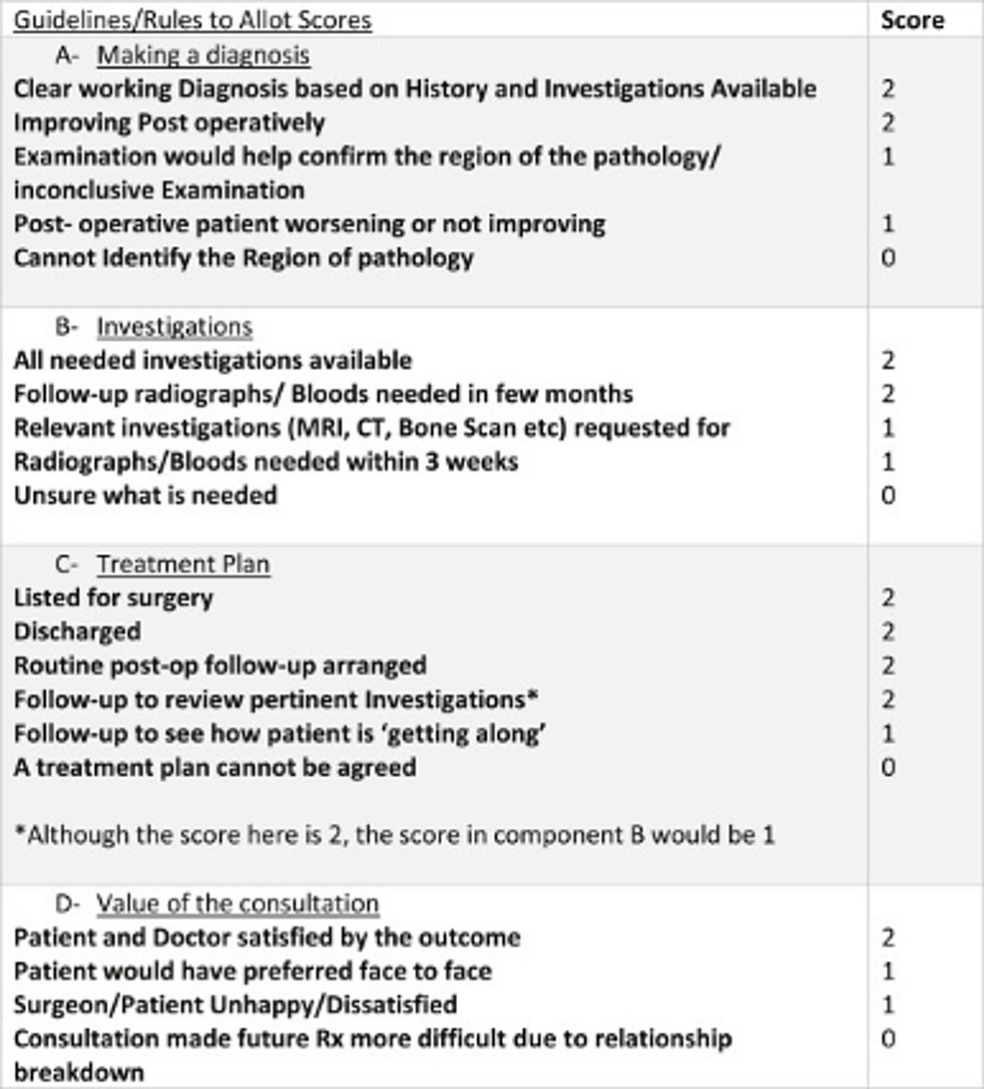
Guidelines for scoring each component of the score

All letters were scored using the ACLS guidelines (Figures 1-2). All data were entered into a Microsoft Excel sheet (Microsoft Corp, Redmond, WA) to give us a final score for each parameter scored. A detailed analysis was performed to see how telemedicine performed against face-to-face consultations in various trauma scenarios.

## Results

**Figure 3:**
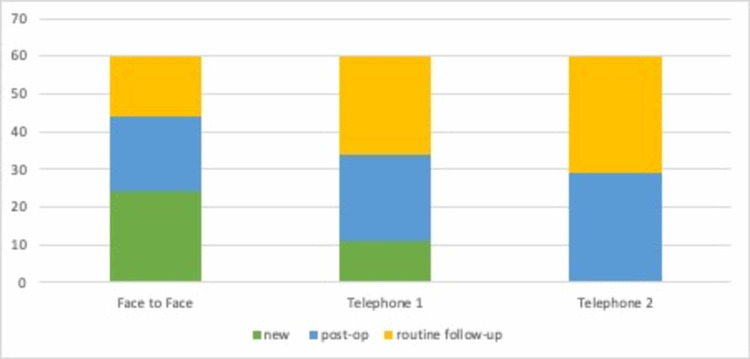
Distribution of patients across all consultation groups

The physical appointment cohort comprised 60 patients; 24 were new patients and 36 follow-up consultations, of which 20 were postoperative patients. In comparison, the first group of telephone consultations only had 11 new patients, and 23 of the patients were postoperative patients. The second group had no new patients and 29 of the patients were postoperative. See Figure 3.

**Figure 4:**
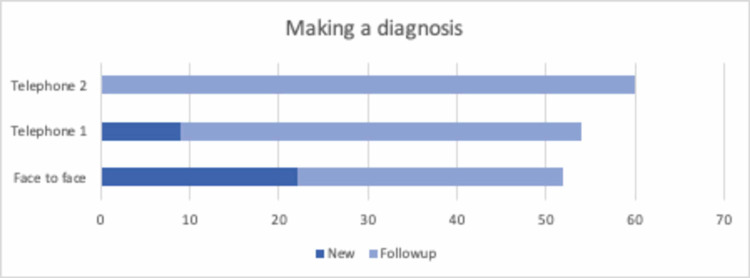
The ability to make a clinical diagnosis across all consultations

There was a clinical working diagnosis in 87% of the face-to-face appointment patients, 90% of the first group telephone appointment patients and 100% of the second group of telemedicine patients (Figure 4). The relative risk of failing to make a diagnosis with a telephone consultation as compared to a physical appointment was 0.388, 95% confidence interval 0.1407 - 1.0673, which was not statistically significant. However, the relative risk increased to 2.18 amongst new patients, which, however, also did not reach statistical significance.

Fifty-five per cent (55%) of patients seen in the face-to-face clinic already had required investigations available (score 2), 8% had urgent relevant investigations arranged during the same appointment, 33% had investigations required in weeks and 3% in months. In contrast, 80% of patients with telephone appointments had required investigations available, 18% needed follow-up investigations in months and only 3% needed urgent investigations to be arranged immediately.

**Figure 5:**
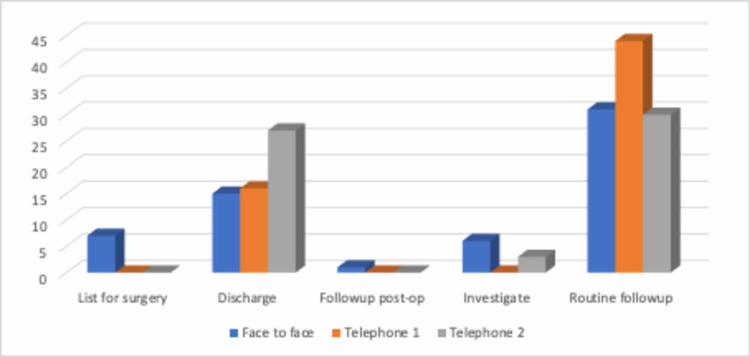
Outcomes from the consultations across all groups

Across the three cohorts, only the face-to-face clinic group had 11.7% of the patients listed for a surgical procedure; the telephone consultation group had no patients listed for surgery. The discharge rate from the face-to-face clinic was about 25%, with 10% of patients sent for further investigations and 50% booked for routine follow-up appointments. In comparison, the initial telephone appointment group had a similar discharge rate to the face-to-face appointment cohort (26.7%) but a significant tendency towards routine follow up (73%). The second telephone appointment group fared better than the other two groups with only 50% routine follow-up (matching face-to-face clinics), and a 45% discharge rate, with the remaining patients sent for further investigations. See Figure 5. There was no record of any patient or doctor dissatisfaction with the consultations across all groups.

The mean ACLS score for the face-to-face clinic letters was 6.7; the letters from both groups of telemedicine appointments scored better, the first group scoring 7.1 and the second 7.45. The combined telemedicine appointment letters scored better than the face-to-face clinic letters (7.275). An independent-samples unpaired t-test was conducted to compare the ACLS mean scores for the face-to-face appointment and telemedicine clinic letters. There was a statistically significant difference in the mean scores for face-to-face and telemedicine letters (p < 0.001) (Table 1). Specifically, our results suggest that telemedicine consultations performed better than face-to-face consultations according to the ACLS letter scoring system.

**Table 1:** Statistical analysis of the Ashford Clinic Letter Scores (ACLS) from the different clinics

	Face to face	Telephone 1	Telephone 2	Combined Telephone
Median	7	7	7	7
Mean Score	6.7	7.1	7.45	7.275
Mode	8	7	7	7
Range	4	3	1	3
S.D	1.151147794	0.752397299	0.501692052	0.660563803

## Discussion

Our study shows that according to the Ashford Clinic Letter Scoring system, telemedicine is an effective alternative for treating patients with orthopaedics injuries. According to Caldwell (2019), the question that the clinician is trying to answer during a consultation is ‘What is the diagnosis?’ so that appropriate investigations, treatment and follow-up can be planned [6]. In our study, it is interesting to note that 90% of the first group of telemedicine consultations had a working diagnosis and 100% in the second group as compared to 87% for the face-to-face appointment group. These results suggest that clinicians got more confident in making a clinical diagnosis in the virtual appointment cohort with time. Alternatively, as these groups were not matched for diagnoses, clinical history and or mechanism of injury, it could suggest clinical heterogeneity in terms of complexities of the referrals. However, it is expected in the setting of ‘fracture clinics’ to be able to make a diagnosis in most if not all the patients based on the history and radiology reports.

It is important to note that there were no new patients in the second group of telephone clinic patients. This could suggest that during the peak of the COVID-19 pandemic, there were fewer presentations of orthopaedic trauma to the Accident and Emergency (A&E) department requiring referral to the clinic, which could have been secondary to the lockdown. We did not investigate how many orthopaedic injuries were seen during this time in the A&E department, which could have helped confirm whether there were fewer presentations. It is equally essential to note that new patients could have been pre-triaged to the face-to-face clinic, however, the volumes of new patients seen in the physical appointment clinics during this period were significantly lower than during pre-COVID times.

Most telemedicine patients (80%) already had investigations available on the system from a previous visit, as the majority were follow-up patients. As expected, the face-to-face appointment group endured more emergency (during the same appointment) or urgent (within weeks on follow-up) investigations. This could suggest that face-to-face appointment patients may undergo unnecessary repeat radiographs or further investigations. As our study was limited to purely assessing the ‘quality’ of the consultation using the ACLS, we did not investigate the indications for these additional investigations and whether they were appropriate. On the other hand, having a telemedicine appointment did not impede or delay any relevant clinical investigation, and in most instances (18%), such investigations were only required in a long-term follow-up appointment. These results suggest that patients who do get an initial face-to-face appointment, and/or those that have had an operation can be safely and effectively followed up with subsequent telemedicine appointments once the diagnosis/treatment plan has been established.

The discharge rate from the face-to-face appointment was 25% as compared to the discharge rate of 36% across the combined telemedicine group. This discharge rate is similar to an overall discharge rate, which varies from 33%-60% from virtual fracture clinics [7]. Although we do not run a dedicated virtual fracture clinic in our unit, during this period, we saw a decrease in the number of patients seen in the face-to-face fracture clinics of around 70% as the majority of patients were followed up using telemedicine. This is consistent with the findings of the Glasgow Fracture Pathway, which showed that 55%-67% of ED referrals that are seen in a traditional fracture clinic are appropriate to be followed up in a virtual clinic; additionally, Anderson et al. showed a 65% reduction in face-to-face consultations in a fully implemented telemedicine service [7-8]. As stated earlier, an alternative explanation could be that the lockdown massively reduced the volume of trauma patients presenting to the A&E as suggested by Hampton et al. and, consequently, the number of referrals to the fracture clinic [9].

The mean ACLS scores from the telemedicine appointment letters suggest that telephone consultation for routine fracture clinic and post-operative follow-up of trauma and orthopaedics patients is safe, effective and comparable or even superior to face-to-face clinic appointments. Nevertheless, there are inherent weaknesses when a clinical letter is used as a surrogate for a consultation; the most significant being poor documentation, which can only be mitigated by prospective studies [5]. Our results strongly suggest that most fracture clinic patients can be managed safely, and effectively with telemedicine appointments for routine and post-operative follow-up. The advancement in technology and online patient care systems makes it possible for investigations to be ordered or reviewed and plaster change or removal appointments to be remotely requested, maintaining patient satisfaction and ‘hospital distancing’ in the whole process. This has been shown to significantly reduce the number of patients in traditional fracture clinics, save costs, help free up members of staff to support in other clinical areas and ostensibly reduce the risk of hospital-acquired COVID-19 infections [7,10]. In addition, to make the consultation process more rigorous, we can have the consultations via video to aid in the examination of the affected area and be able to witness the patient perform certain movements. However, the caveat is the requirement of having a securely recognised video consultation link with a patient who is able to have access to and use the technology.

## Conclusions

As the COVID-19 pandemic continues, we need to continue to adapt and change our practice accordingly. One important way of doing this with clinics is via telemedicine. We have shown that telephone medicine can provide a valuable diagnosis, allow the organisation of relevant investigations and enable the formulation of an appropriate treatment plan for the patient. Furthermore, the Ashford Clinic Letter Scoring System (ACLS) provides an appropriate, concise way of measuring these parameters objectively. With the pandemic continuing and, in some places, worsening, telemedicine will continue to thrive, to probably become the backbone of clinical practice in trauma and orthopaedics in the future, thereby limiting patient-to-clinician contact and protecting both members of staff and patients. Further studies need to be carried out to evaluate the effectiveness of remote consultations in regard to validated outcome measures scoring patient satisfaction. Ultimately, the future of clinical practice should involve patients electing between telephone and physical consultation, as we have demonstrated both can be equally effective.
